# SARS-CoV-2 Infection as a Determining Factor to the Precipitation of Ischemic Priapism in a Young Patient with Asymptomatic COVID-19

**DOI:** 10.1155/2021/9936891

**Published:** 2021-07-03

**Authors:** Antonio Francesco Maria Giuliano, Marco Vulpi, Francesca Passerini, Antonio Vavallo, Anna Belfiore, Saverio Forte, Vincenzo Ostilio Palmieri, Pasquale Ditonno

**Affiliations:** ^1^Department of Biomedical Sciences and Human Oncology, Clinica Medica “A. Murri”, Università degli Studi di Bari Aldo Moro Facoltà di Medicina e Chirurgia, Piazza Giulio Cesare n°11, 70124 Bari, Bari, Italy; ^2^Department of Emergency and Organ Transplantation, Urology and Andrology Unit II, Università degli Studi di Bari Aldo Moro Facoltà di Medicina e Chirurgia, Piazza Giulio Cesare n°11, 70124 Bari, Bari, Italy

## Abstract

COVID-19 is a disease characterized by respiratory distress, systemic inflammation, multiple organ dysfunction and coagulation disorders, chiefly pulmonary embolism, and deep venous thrombosis. In this case report, we discuss a peculiar case of ischemic priapism in a 36-year-old patient with asymptomatic COVID-19 and no other plausible causes of thrombophilia and/or alternative causes of priapism, as well as discussing possible explanations for such remarkable findings and comparing them to analogous cases recorded in literature. The patient was unsuccessfully treated via cavernous blood aspiration and required several shunting procedures, with no further recurrences and negative testing for pulmonary embolism, deep venous thrombosis, and other causes of thrombophilia.

## 1. Introduction

The current SARS-CoV-2 pandemic constitutes one of the most intense challenges faced by medicine in contemporary times. This novel coronavirus infection causes a unique form of disease (COVID-19) characterized by respiratory distress, systemic inflammation with multiple organ dysfunction, pulmonary embolism, and deep venous thrombosis [1]. Priapism is defined as a prolonged penile erection originating without any concurrent sexual stimulation, persisting for more than 4 hours and despite orgasm [2]. Conventionally, priapism is classified as ischemic, nonischemic, and stuttering, according to the underlying vascular lesions and clinical presentation: ischemic priapism is considered a medical emergency due to potentially irreparable damage leading to erectile dysfunction with fibrosis of the corpora cavernosa.

In this paper, we present an atypical case of priapism occurring in a patient with asymptomatic SARS-CoV-2 infection, whose peculiarities might offer further clarification of the possible role of SARS-CoV-2-induced systemic inflammation and hypercoagulability in the onset of this urologic emergency.

## 2. Case Presentation

A 34-year-old man was admitted to our unit complaining a 36-hour history of painful erection without sexual stimulation; one month prior to admission, the patient tested positive for SARS-CoV-2 infection via nasopharyngeal swab and had since been completely asymptomatic, save for persistent anosmia. The patient denied any history of pharmacological treatment or substance abuse and further questioning revealed no known risk factors for priapism; vital signs at admission were acceptable (BP: 165/85 mmHg, HR: 78 bpm, and body temperature 37°C). Low flow priapism was confirmed when cavernosal blood gas analysis showed hypoxia, hypercapnia, and acidosis. Two penile blood aspiration and intracavernous phenylephrine injection (diluted in normal saline to a concentration of 100–500 *μ*g/mL and 1 mL given every 3–5 minutes, up to a maximum dosage of 1 mg) attempts proved unsuccessful, and a 14-G needle spongio-cavernosal shunt failed. Therefore, a bilateral T-shunt procedure was performed using a size 10 blade scalpel placed vertically through the glans until fully within the corpus cavernosum. The corpora-glandular shunt was completed by the retrograde insertion of a size 7 Hegar dilator into the distal end of each corpus cavernosum through the original glandular incision and blood evacuation by manual compression of the penis sequentially from a proximal to distal direction. The patient never required supplemental oxygen for the entirety of his hospital stay. A contrast-enhanced chest and pelvis CT scan and a lower extremity venous Doppler study were performed, with no signs of parenchymal involvement or pulmonary embolism nor any evidence of deep venous thrombosis, respectively. Further testing for other plausible causes of thrombophilia (such as factor V Leiden, antithrombin III deficiency, sickle-cell disease, hyperhomocysteinemia, and lupus anticoagulant) or alternative causes of priapism returned negative results. Penile tumescence gradually resolved during the next two days, and the patient was discharged on post-op day six, following complete remission of symptoms and with no need for anticoagulation.

At three-month follow-up, the patient reported complete erectile dysfunction with no further relapses of priapism; phosphodiesterase type 5 inhibitors were not prescribed since the patient underwent a shunting procedure. An inflatable penile implant was suggested but the patient asked to wait for some spontaneous improvement before undergoing surgery.

Key laboratory testing and blood gas analysis results are reported in Tables 1 and 2, respectively.

## 3. Discussion

Ischemic priapism is the most common form of this disease's presentation, a consequence of an obstruction of the penile venous outflow leading to a stasis of hypoxic blood within the corpus cavernosum akin to a proper compartment syndrome. In our patient, the clinical presentation with painful rigid erection, presence of dark, clotted blood at cavernosal blood aspiration, and blood gas analysis findings strongly supported ischemia-related priapism. SARS-CoV-2 infection has been associated to a unique set of coagulation changes collectively referred to as COVID-19-associated coagulopathy [3]: while the virus itself appears to have no intrinsic procoagulant activity, it has been hypothesized that a diffuse microangiopathy can be mediated via endothelial ACE2 receptors, disrupting natural antithrombotic pathways. A number of cytokines involved in the etiopathogenesis of COVID-19 have a well-known role in both inflammation and thrombosis: IL-6 in particular is linked to multiple such procoagulant pathways, purportedly through tissue factor expression on mononuclear cells [4]. Furthermore, available data heavily stresses IL-6's crucial role in COVID-19 pathophysiology [5] as well as in murine models investigating ischemic priapism [6].

Comparison with other literature reported cases of priapism in SARS-CoV-2-affected patients revealed several key differences: our patient's age and asymptomatic COVID-19 with an absence of known comorbidities seem to starkly contrast with the clinical profile noted in the three available reports, namely, older age, severe COVID-19, and admission to an Intensive Care Unit [7]. Notably, administration of anesthetics such as propofol and patient's exitus was recorded in two cases [8, 9]: previous case reports [10] suggest that propofol can cause ischemic priapism, with the work of Vesta et al. [11] confirming a strong association of propofol to priapism with rechallenge. In all three cases, priapism was managed conservatively or by intracavernosal injection of the sympathomimetic agents. Similarities between the reports however seem to corroborate the hypothesis of SARS-CoV-2 playing a role in promoting penile thrombosis regardless of COVID-19 severity and the onset of cytokine storms, both due to endothelial damage and through the prothrombotic action of cytokines, chiefly IL-6.

Our case, to our knowledge, represents the first report of priapism in mild COVID-19 without necessity of intubation and mechanical ventilation, managed by T-shunt procedure with potential permanent erectile dysfunction.

## 4. Conclusion

All data considered, priapism could represent a very rare yet plausible complication in the context of SARS-CoV-2 infection, regardless of symptoms, disease severity, patient's age, and concurrent risk factors. In particular, the role of a multidisciplinary evaluation involving emergency medicine, internal medicine, and urology specialists appears pivotal in ensuring swift detection and prompt, aggressive management of such an occurrence, since this condition is not commonly seen in clinical practice and may go unrecognized with potentially irreversible consequences. ACE2-induced microangiopathy and cytokine-activated thrombosis, especially IL-6-mediated, might be crucial in fully understanding the link between COVID-19 and thrombotic events, as well as potentially offering novel diagnostic and therapeutic approaches on the matter. Further data is auspicial in order to provide a clearer insight.

## Figures and Tables

**Table 1:** Key laboratory tests at admission. MCV: mean cellular volume; ESR: erythrocyte sedimentation rate; CRP: C-reactive protein; n/a: not applicable.

	Patient	Laboratory standard
Erythrocytes (×10^6^/*μ*L)	3.07	4.54-5.78
Leukocytes (×10^3^/*μ*L)	8.61	3.7-9.7
Hemoglobin (g/dL)	9.3	13.3-17.2
Hematocrit (%)	26.9	38.9-50.9
MCV (fl)	87.4	81.2-94
Neutrophils (%)	68.9	42.9-78.4
Eosinophiles (%)	4	0.3-6.2
Basophiles (%)	0.5	0.3-1.3
Lymphocytes (%)	20.9	14.1-45.8
Monocytes (%)	5.7	3.3-9.2
Platelets (×10^3^/*μ*L)	363	179-373
Urea (mg/dL)	28	15-38
Bilirubin (mg/dL)	0.32	0.2-1
Glucose (mg/dL)	95	<100
Glycated hemoglobin (mmol/mol)	36	20-42
Prothrombin time (INR)	1.01	n/a
Fibrinogen (mg/dL)	429	200-400
Activated partial thromboplastin time (ratio)	0.89	<1.20
D-dimers (*μ*g/L)	5855	<500
Homocysteine (mmol/L)	21.7	3.2-10.7
Antithrombin III (%)	116	80-130
ESR (mm/h)	69	1-15
CRP (mg/L)	37.5	<2.9
Presepsin (pg/mL)	351	20-200
IL-6 (*μ*g/L)	76.4	0-7
Procalcitonin (ng/mL)	1.19	0-0.05

**Table 2:** Arterial and penile blood gas analysis. n/a: not applicable.

	Arterial blood gas	Cavernosal blood gas	Laboratory standard
pH	7.51	7.47	7.35-7.45
pCO_2_ (mmHg)	35	33	35-45
pO_2_ (mmHg)	83	65	80-100
Na^+^ (mmol/L)	135	139	135-150
K^+^ (mmol/L)	3.1	2.7	3.4-5.0
Cl^−^ (mmol/L)	103	111	98-107
Ca^2+^ (mmol/L)	1.12	1.03	1.15-1.27
Glucose (mg/dL)	108	95	74-106
Lactates (mmol/L)	0.8	1.2	0.4-2.0
P/F	395	309	>400
HCO_3_^−^ (mmol/L)	28.6	25.2	22-28
Oxygen saturation (%)	98.7	94.8	>94%
FiO_2_ (%)	21	21	n/a

## Data Availability

The data used to support the findings of this study are included in the paper.
